# A Flexible and Wearable Human Stress Monitoring Patch

**DOI:** 10.1038/srep23468

**Published:** 2016-03-23

**Authors:** Sunghyun Yoon, Jai Kyoung Sim, Young-Ho Cho

**Affiliations:** 1 NanoSentuating Systems Laboratory, Cell Bench Research Center Korea Advanced Institute of Science and Technology (KAIST), 271 Daehak-ro, Yuseong-gu, Daejeon 305-701, Republic of Korea

## Abstract

A human stress monitoring patch integrates three sensors of skin temperature, skin conductance, and pulsewave in the size of stamp (25 mm × 15 mm × 72 μm) in order to enhance wearing comfort with small skin contact area and high flexibility. The skin contact area is minimized through the invention of an integrated multi-layer structure and the associated microfabrication process; thus being reduced to 1/125 of that of the conventional single-layer multiple sensors. The patch flexibility is increased mainly by the development of flexible pulsewave sensor, made of a flexible piezoelectric membrane supported by a perforated polyimide membrane. In the human physiological range, the fabricated stress patch measures skin temperature with the sensitivity of 0.31 Ω/°C, skin conductance with the sensitivity of 0.28 μV/0.02 μS, and pulse wave with the response time of 70 msec. The skin-attachable stress patch, capable to detect multimodal bio-signals, shows potential for application to wearable emotion monitoring.

Continuous psychological stress monitoring in daily life is important to reduce the negative influences^1^,^2^,^3^,^4^,^5^ of psychological stress to Human society and health. Stress affects human society by increasing suicide^1^ and reducing work efficiency^2^. Also, stress leads many kinds of diseases^4^ such as shock, cardiovascular diseases, neuropsychiatric diseases, gastro intestinal diseases, and even tumors. According to the American Psychological Association, “job stress is estimated to cost U.S. industry $300 billion a year in absenteeism, diminished productivity, employee turnover and direct medical, legal and insurance fees^5^”. Therefore, monitoring psychological stress level is important to both patients and non-patients for better life.

There are three conventional methods to measure psychological stress: self-report^6^, body fluid analysis^7^, and multimodal physiological data analysis^8^. The self-report method is hard to monitor human stress consistently due to the lack of standards for stress status^7^. The body fluid analysis cannot measure stress continuously. However, the multimodal physiological data analysis is the suitable method to monitor physiological stress in daily life because it measures psychological stress consistently and continuously.

Lots of the researches have studied about how to measure human psychological stress using physiological data: relationships between stress and physiological data^9^,^10^,^11^,^12^,^13^,^14^,^15^, improvement of stress detecting accuracy using machine learning algorithms^16^,^17^,^18^,^19^,^20^, long term stress monitoring^21^,^22^ in real daily life, and designing stress monitoring device^23^,^24^,^25^,^26^,^27^,^28^,^29^,^30^,^31^. Based on the previous studies, it is proved that the physiological data for stress monitoring are responses of human autonomous nervous system (ANS)^10^. The ANS is excited by various kinds of stressors (origins of the stress), such as audial^32^, visual^33^, thermal^34^,^35^, working^8^,^36^, and exercising^37^,^38^ stimulations. Therefore ANS signal analysis enables the various kinds of the stress analysis. Among ANS responses, the skin temperature^11^,^26^, skin conductance^11^,^26^,^28^, and arterial pulsewave^11^,^23^,^26^,^28^ signals are required for the comprehensive multimodal physiological data analysis. The stress signals of skin temperature, skin conductance, and pulsewave are dependent on the stimulation time and categorized in two types: acute stress (response under 3 seconds stimulation)^11^ and chronic stress (response over 3 seconds stimulation)^39^. Peripheral skin temperature shows negative relationship^23^,^40^ with the chronic stress level. Skin conductance on palm and volar part of the wrist has positive relationship^11^ with both of the chronic and the acute stress levels. For stress characterization, the arterial pulsewave signals is required to be transformed into the human heart rate variability (HRV)^39^. HRV is different with heart rate (HR). HR is a number of the peaks of the arterial pulsewave within 1 minute. The HRV is defined by the frequency analysis of the time interval between the peaks of arterial pulsation. The HRV represents the chronic stress level^10^ and also individual’s stress vulnerability^12^.

The conventional researches about stress monitoring devices are focused on designing completed systems using commercial physiological sensors; thereby having bulky size^41^ to be worn or carried for daily time use. For example, a glove-type device^23^ uses the multiple hard sensors assembled on a single layer, resulting in a poor wearing comfort due to low flexibility and large skin contact area. On the other hand, though good wearing comfort, a watch type daily time stress monitoring device^31^ is not able to comprehensive stress analysis by measuring only skin conductance. Therefore, wearing comfort enhanced comprehensive stress monitoring device development is necessary.

In this study, we invent a human stress monitoring patch with small skin contact area and high flexibility to enhance wearing comfort of the patch. The skin contact area minimization is achieved by the multi-layer integration of the skin temperature sensor and the skin conductance sensor on the patch type pulsewave sensor. The low flexibility of the conventional sensors is mainly due to the hardness of pulsewave sensor. In this study, we enhance the flexibility of the pulsewave sensor by replacing the rigid piezoelectric material and support^42^ by the flexible flexible piezoelectric material of Poly(vinylidene fluoride-trifluoroethylene) [P(VDF-TrFE)]^43^,^44^,^45^,^46^,^47^ on the flexible polyimide^48^,^49^,^50^,^51^,^52^ support. We use the polyimide as the support membrane not only due to high flexibility but also due to high chemical resistance^40^. We use P(VDF-TrFE) as flexible piezoelectric materials with high mechanical strength and biocompatibility^43^.

The flexible piezoelectric material of P(VDF-TrFE), however, has the lower piezoelectric coefficient (−38 pC/N)^44^, resulting in the lower pulsewave sensing efficiency compared to that of rigid piezoelectric material, such as PZT-4 (289 pC/N)^44^. In the present patch, we form windows on the polyimide support membrane underneath the P(VDF-TrFE) membrane to induce the higher mechanical stress on the piezoelectric material for the higher pulsewave sensing sensitivity. The windows on the polyimide support provides the additional effect of flexibility increase, desirable for conformal skin contact.

We develop a novel structure and process for the flexible multilayer stress patch, where a novel silver inter-layer electrode is prepared between the piezoelectric and support membranes. The silver electrode layer is specially designed to perform triple functions: 1) an etch stop for protecting the piezoelectric membrane during the polyimide window etching process using KOH, Ethanol, and DI water (1:8:2) mixture^52^, 2) an electrode layer for piezoelectric poling process; 3) an electrode layer for sensing the pulsewave signals generated by piezoelectric material. The simple fabrication process developed for the flexible multi-layer stress patch is described in the method section.

The flexible multi-layer patch (see Fig. 1), integrated with the skin temperature sensor, the skin conductance sensor on the pulsewave sensor, results in skin contact area reduction and patch flexibility increase; thereby enhancing the wearing comfort, required for wearable or skin-attachable stress monitoring by human ANS monitoring.

## Results

### Design and Principles

**Figure 2:**
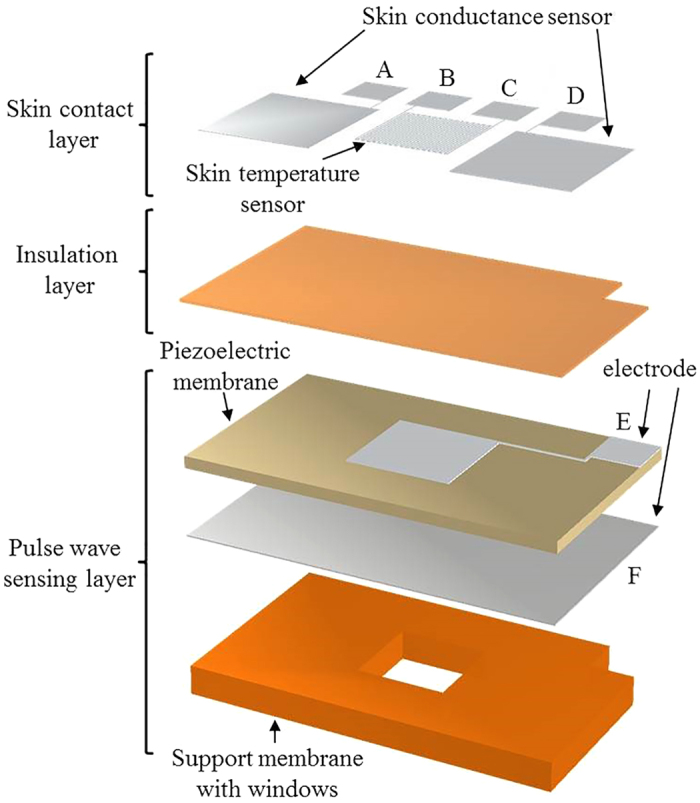
Layers of the flexible human stress monitoring patch.

The stress monitoring patch consists of three layers (see Fig. 2): a skin contact layer, an insulation layer, and a pulsewave sensing layer. The skin contact layer, making direct skin contacts, contains the skin temperature sensor and skin conductance sensor both using aluminum electrodes. The insulation layer of parylene-C provides the electrical isolation between the skin contact layer and the pulsewave sensing layer. In the pulsewave sensing layer, the silver electrode is sandwiched between the piezoelectric membrane and the polyimide support membrane with windows.

Dimensions and performance of the patch are design to detect the Human physiological signals, including skin temperature, skin conductance and pulsewave. The human peripheral skin temperature and skin conductance are changed in the range of 30–40 °C^53^ and 2–20 μS^9^, respectively. Human pulsewave is strongly depending on the Human states, such that the systolic-diastolic pressure difference is varying in the range of 40–120 mmHg at the frequency of 50–200 BPM (0.83–3.66 Hz)^54^ between Human resting state and exercising state.

**Figure 3:**
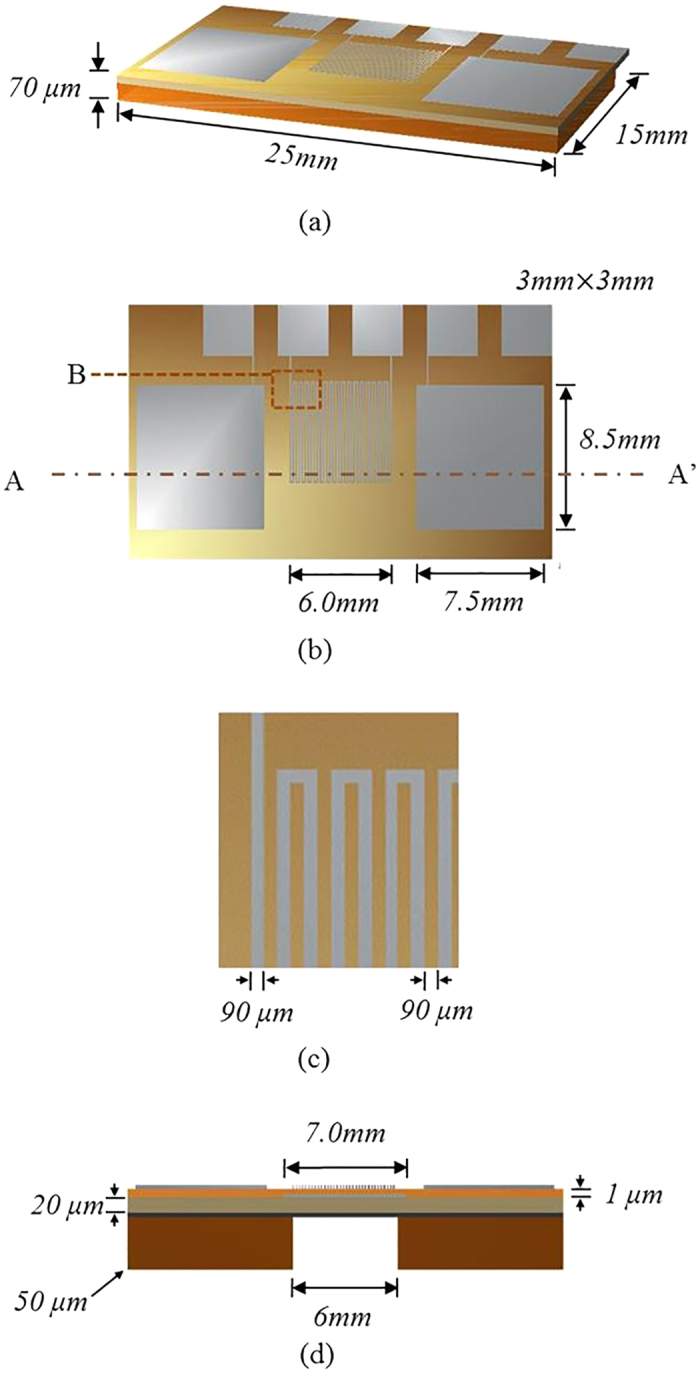
The flexible human stress monitoring patch: (**a**) perspective view; (**b**) top view; (**c**) enlarged view of B in Fig. 3b; (**d**) cross-sectional view along A-A’ of Fig. 3b.

**Figure 4:**
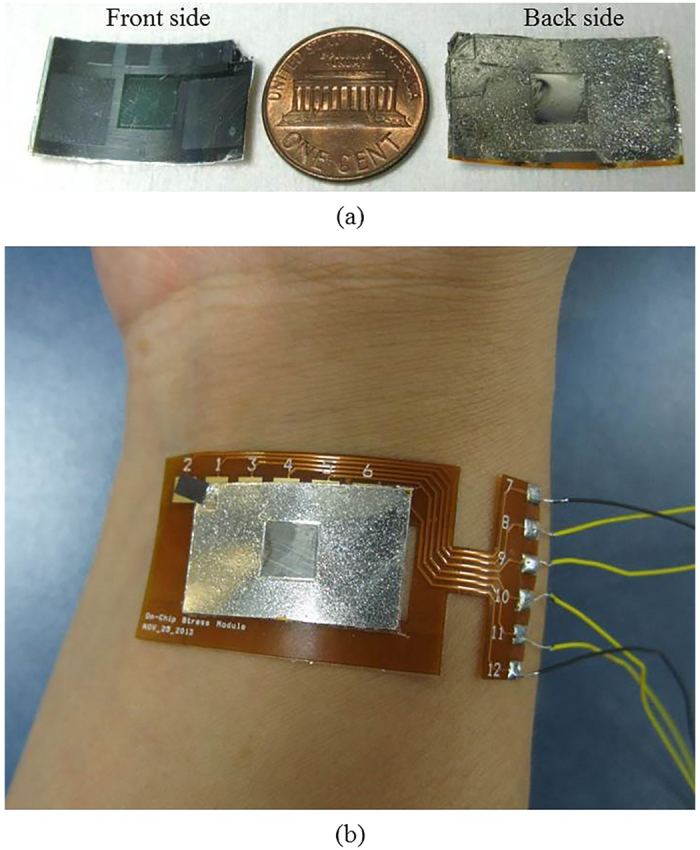
The fabricated human stress monitoring patch: (**a**) the front and back-side of the patch, compared to a US penny; (**b**) the patch attached to human wrist.

The individual sensors of the patch are designed to detect the human physiological signals. The pulsewave sensor detects piezoelectric output voltage generated by the mechanical stress of the piezoelectric membrane from the pulsation of radial artery at human wrist. The window of the pulsewave sensor is designed as 6 mm × 6 mm, capable to cover the radial artery sufficiently by having twice larger width than the radial artery diameter of 2.19–3.01 mm^55^. The thickness of the piezoelectric membrane of the pulsewave sensor is decided as 20 μm, considering the maximum poling thickness^43^ for P(VDF-TrFE). The commercial 50 μm-thick Kapton film (Kapton^®^ HN, Dupont™) is used for the polyimide support membrane. The skin conductance sensor adapts bipolar recording method^9^ to measure the electric current change due to conductance change for the constant electric voltage supplied to two electrodes of the sensor. The electrodes of the skin conductance sensor requires large skin contact area to cover as many numbers of sweat glands as possible. Thus each electrode is designed as 7.5 mm × 8.5 mm to have skin contact area of 63.75 mm^2^, larger than the single electrode area (6.36 mm^2^) of a commercial skin conductance sensor (HK-228s, iWorx). The two electrodes are located across the window on the polyimide membrane. The skin temperature sensor for resistance temperature detection (RTD) is made of aluminum. The RTD resistance is designed as 125 Ω, require for Human skin temperature detection^53^. To minimize the skin contact area of the patch, the RTD electrode, having the size of 90 μm linewidth and gaps (see Fig. 3c), is located on the insulation layer aligned with the window. The six electric contact pads are designed as 3 mm × 3 mm for easier electric wiring. The skin conductance sensing electrodes, the RTD electrode, and the electric contact pads are fabricated simultaneously by patterning 0.5 μm-thick aluminum on the insulation layer. Therefore, the overall size of the flexible human stress monitoring patch (see Fig. 4) is decided as 25 mm × 15 mm × 72 μm (see Fig. 3), having the skin contact area of 3.75 cm^2^. The specific fabrication process of the patch is indicated in the method section.

### Theoretical Simulation

We analyze the effects of the windows to the patch flexibility and the pulsewave sensing sensitivity of the two prototypes, the patch with windows (Type A) and that without windows (Type B).

The flexibility of Type A and Type B are analyzed in terms of bending stiffness. For the fixed and free end conditions, the bending deflections of the energy harvesters for the free end load *P*^56^ are given by


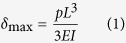


where *δ*_*max*_ is the maximum free end deflection, *P* is the free end force, *L* is the patch length, and *EI* is the bending stiffness of the patch. For *P* of 30 μN, the maximum deflections of Type A and Type B are estimated from COMSOL Multiphysics^®^ and the bending stiffness of Type A and Type B are obtained by Eq. (1) as 89.21 μNm^2^ and 95.51 μNm^2^, respectively.

**Figure 5:**
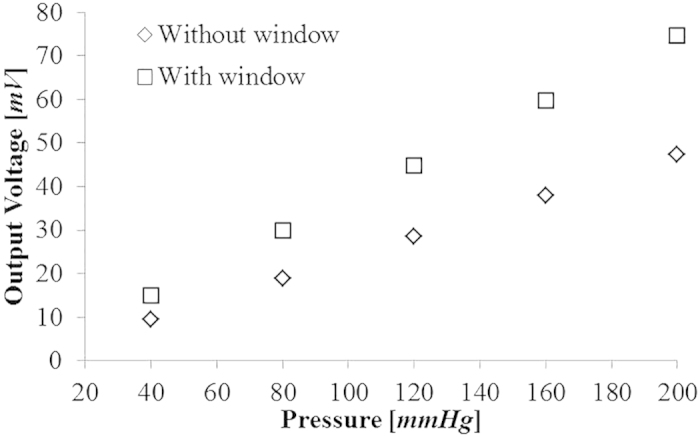
Theoretical results of the pressure-dependent output voltage of the pulsewave sensors, with and without window, respectively.

For pulsewave sensing sensitivity comparison, the piezoelectric output voltages of Type A and Type B are estimated for the five different pressures of 40, 80, 120, 160 and 200 mmHg within the range of pressure difference between human systolic and diastolic arterial pressure. The mechanical stress on the piezoelectric membrane are estimated by COMSOL Multiphysics^®^ for the five different pressures. The piezoelectric output voltage (see Fig. 5) of the pulsewave sensor is obtained using MATLAB on the baisis of the obtained mechanical stress. The pulsewave sensing sensitivities of Type A and Type B are shown as 0.373 mV/mmHg and 0.237 mV/mmHg, respectively. The theoretical results indicate that Type A with window shows the 6.60% increase of the patch flexibility and 57% increase of the pulsewave sensing sensitivity compared to Type B without window.

### Experimental Characterization

The stress monitoring performance of the three individual sensors integrated in the patch are experimentally characterized in the human physiological ranges.

#### Skin Temperature Sensor

**Figure 6:**
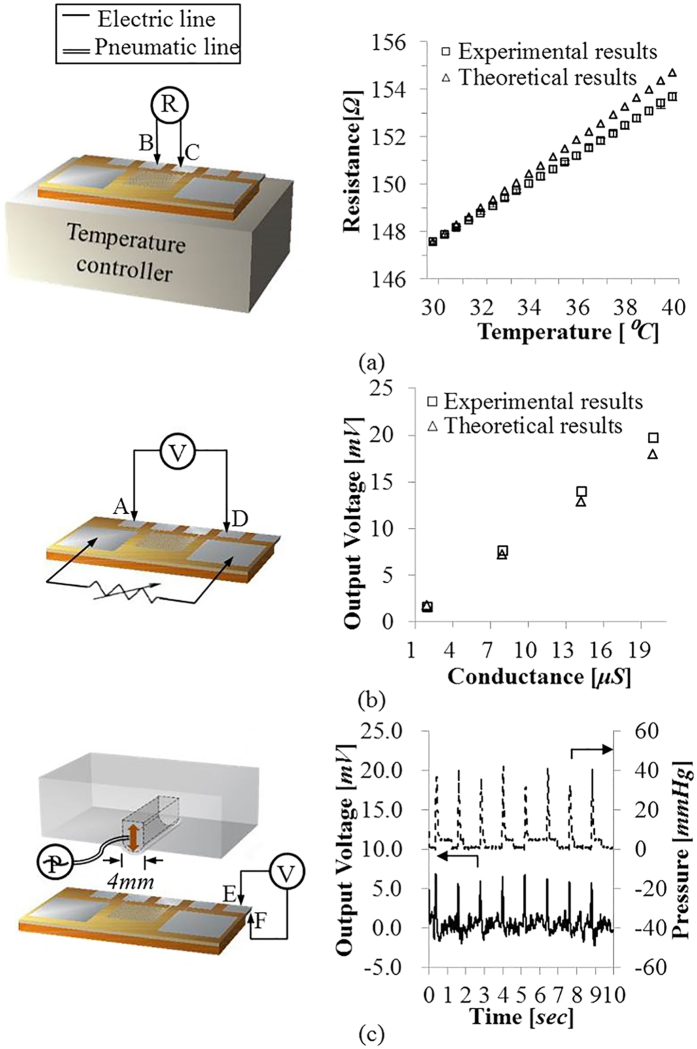
Experimental setup and performance of the flexible human stress monitoring patch: (**a**) Temperature-dependent resistance of the skin temperature sensor; (**b**) Conductance- dependent voltage of the skin conductance sensor; (**c**) time–dependent voltage of the pulsewave sensor for the input pressure pulse of 40 mmHg at 50 BPM.

The fabricated skin temperature sensor is characterized in the temperature range of 30–40 °C with 0.5 °C increments, using a temperature controller (TC01, Multichannel systems MCS GmbH). The skin temperature sensor nonlinearity and sensitivity (see Fig. 6a) are measured as 0.021% and 0.31 Ω/°C, respectively.

#### Skin Conductance Sensor

The voltage output of the skin conductance sensor is measured for the conductance change of 2–20 μS. The skin conductance sensor has showed the nonlinearity, resolution, and sensitivity (see Fig. 6b) as 0.447%, 0.02 μS, and 0.28 μV/0.02 μS, respectively, within the human skin conductance range.

#### Pulsewave Sensor

The pulsewave sensor performance is measured (see Fig. 6c) using an artificial artery actuator. The artificial artery actuator is fabricated by the bonding of PDMS membrane and PDMS structure having the 4 mm-wide artificial radial artery, using a plasma bonding technology. The fabricated patch is attached to the actuator, where the input pressure pulse of the 40–120 mmHg is provided at the frequency of 50–220 BPM. Figure 6c demonstrates the output signal of the pulsewave sensor is correlated well with the amplitude of 40 mmHg and the frequency of 50 BPM of input pressure pulse equivalent to the arterial pulsewave’s pressure difference amplitude and frequency at human resting state.

**Figure 7:**
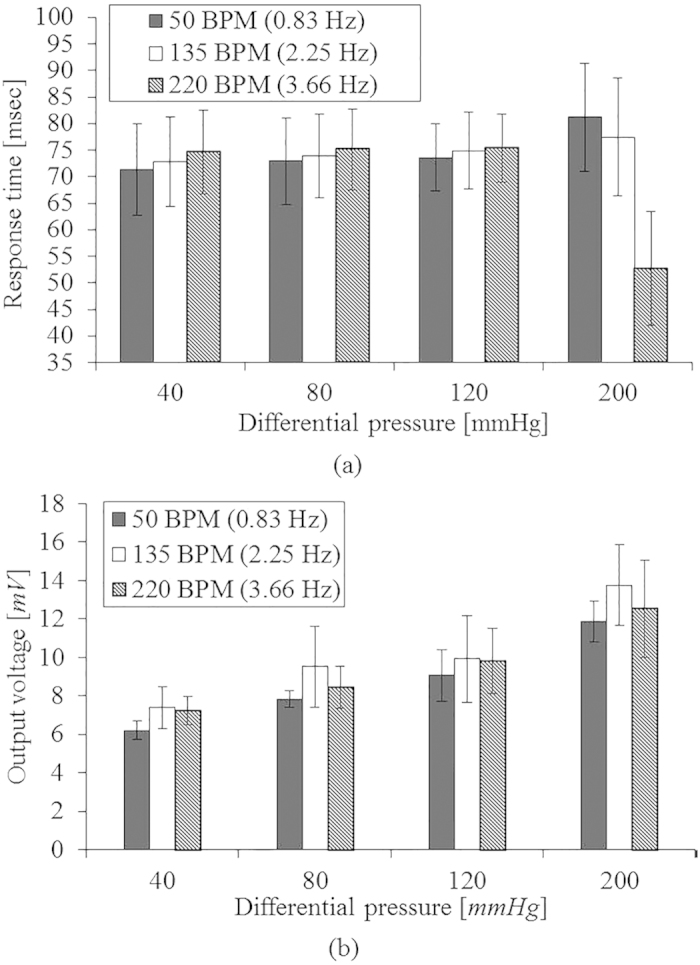
Performance of the pulsewave sensor for varying differential pressure of heart beat (40 ~ 120 mmHg) depending on the heart rate of 50 BPM, 135 BPM and 220 BPM: (**a**) time delay; (**b**) output voltage.

The time delay of the pulsewave sensor (see Fig. 7a) is maintained as 70.00 ± 7.42 msec. The pulsewave sensor sensitivities (see Fig. 7b) at different frequencies of 50 BPM, 135 BPM, and 220 BPM are 0.35 mV/mmHg, 0.38 mV/mmHg, and 0.33 mV/mmHg, respectively. In the stress measurement application, the time delay is more important factor than the output voltage amplitude or sensitivity because HRV is directly related with time interval between peaks of the output voltage signal of the pulsewave sensor, not amplitude or sensitivity. Therefore the fabricated pulsewave sensor integrated in the patch is suitable for human stress monitoring with HRV, though the experimental output voltage and sensitivity of the pulsewave sensor are lower than that of the simulation result. All the experimentally verified performances of the patch are listed in Table 1, demonstrating feasible performance for human stress monitoring applications.

## Discussion

The wearing comfort^57^,^58^,^59^ of wearable devices is decided by; 1) the number of wearing devices; 2) device’s size and weight; 3) flexibility of the device. The above mentioned items are important factors to evaluate the subjective wearing comfort objectively. In other words, people feel the wearable devices are comfortable when they wearing the less number of devices, small and light devices, and highly flexible devices. The present patch satisfies not only small but also other two standards even though the present study didn’t perform subjective comfortable test. The present patch composed of the three sensors are integrated in a single multi-layer structure to reduce the number of devices and the skin contact area of the patch. As a result, the skin contact area of the fabricated multi-layer patch (3.71 cm^2^) is as small as the 1/125 of that of the conventional single layer multimodal stress monitoring device (451 cm^2^)^28^. To increase the flexibility of the patch, we design the flexible pulsewave sensor using the polyimide support membrane and the P(VDF-TrFE) piezoelectric membrane. For additional flexibility enhancement, we design the windows on the polyimide membrane, thus reducing the patch’s bending stiffness in the amount of 6.60%, theoretically. In conclusion, the patch enhances wearing comfort by the multi-layer integration of the three sensors and the windows fabrication on its support membrane.

Stress monitoring ability in daily time is decided by not only a wearing comfort but also a life time of the patch. The skin temperature sensor and the skin conductance sensor life time is related with aluminum corrosion time. Aluminum corrosion rate^60^ changes in the range of 0.06–0.7 μm/year according to surrounding environment. Thus, the skin temperature sensor and the skin conductance sensor life time is estimated in the range of 8–100 months, considering the 0.5 μm-thick electrodes. The pulsewave sensor life time is decided by retention time of the piezoelectric P(VDF-TrFE). The retention time of P(VDF-TrFE) fabricated in a same method of the present patch is known as 0.8 × 10^6 ^seconds^61^. Therefore, considering life times of each sensor integrated in the patch, the patch life time is estimated as 9 days.

The present patch is weakly affected by external environment changes such as temperature and muscle contractions. In the range of environment temperature (0–30 °C), the temperature of the skin temperature sensor on the patch reach to the body temperature within maximum 52.5 msec. Therefore the present patch is weekly affected by the environmental temperature change period over 52.5 msec.

To estimate the influence of the external temperature change to the skin temperature sensor of the patch, we theoretically calculate the response of the skin temperature sensor on the patch for the external temperature and skin temperature change, using FEA (Finite Element Analysis) with Comsol Multiphysics^®^. We calculate the temperature of the aluminum electrode of the skin temperature sensor, considering 5 different peripheral skin temperature (30, 32.5, 35, 37.5, 40 °C) and 4 different external temperature (0, 10, 20, 30 °C). The initial temperature of the patch is decided as 27 °C. The temperature of the electrode is transiently calculated in the range of 0 to 100 msec with 0.5 msec interval. Table S1 shows the time to reach the saturation temperature of the electrode by the skin temperature and external temperature. Table S2 shows the electrode’s saturation temperature by the skin temperature and the external temperature. Figure S1 shows the time response of the temperature difference between the skin and the electrode at each external temperature. The skin temperature sensor of the patch has less than 0.5 msec of time constant, theoretically. In all the cases, the temperature of the skin temperature sensor is saturated at the skin temperature, therefore being dominantly affected not by external temperature but by skin temperature.

Also, muscle-contractions can interfere to pulsewave sensor, occurring motion artifacts. The motion artifacts on pulsewave signal is able to be removed by signal processing^62^,^63^,^64^,^65^ without using additional motion sensors. HRV is able to be estimated from the pulsewave signal^62^ by significantly reducing the influence of motion artifacts. Therefore, the present patch is able to measure pulsewave for human stress monitoring with additional signal processing.

The integrated patch enables the continuous measurement and the comprehensive analysis of human psychological stress by quantitative and continuous measurement of skin temperature, skin conductance, and pulsewave in the human physiological range. Moreover, the application of the patch can be expandable to sense certain dimensions of human emotion. The patch categorizes the 4 kinds of human emotions (surprise, anger, stress, and sadness) based on singular vector machine (SVM) algorithm^17^. Therefore, the patch enhanced wearing comfort has potentials to human emotion sensor for daily use.

The novelty of the present patch is compact size and high flexibility; thereby, easily built into the commercial wearable and mobile devices. The present patch has applications for not only patients but also non-patients by monitoring ANS. For patients, the present patch can alert or notice the potential risks related with ANS to doctors for medical patients monitoring, using communication modules of the wearable devices. Also, the present patch alert the emergency situations like heart attack to rescue team. For non-patients, the present patch can provide wellness information required to individual users.

In summary, we design, fabricate, and characterize the human stress monitoring patch composed of a skin temperature sensor, a skin conductance sensor and a pulsewave sensor. The wearing comfort of the patch is enhanced by the windows fabrication and the multi-layer integration using the silver inter-layer electrode. We experimentally show the individual sensors of the patch have proper performances for the comprehensive analysis of human physiological stress. The small size (24.7 mm × 15.0 mm × 70 μm) and light weight (49.5 mg) of the stress monitoring patch with improved wearing comfort has potentials for multimodal bio-signal monitoring applications and emotion sensing applications using mobile electronics and wearable devices.

## Methods

### Fabrication Process

In the fabrication process of the stress monitoring patch increasing wearing comfort, the windows formation by patterning polyimide is a key process to increase the piezoelectric sensitivity of the pulsewave sensor with the patch flexibility. The windows fabrication on the polyimide support membrane, also, enables the electric contact of the silver inter-layer electrode sandwiched between the polyimide and the P(VDF-TrFE) membranes. The conventional polyimide patterning processes use reactive ion etching (RIE)^66^ or chemical etching process^51^,^52^ with the polyimide etchant of KOH-ethanol-DI water mixture. The conventional RIE polyimide etching process causes thermal damages. The conventional chemical etching process leaves highly viscous polyimide residue which hinders electrical contacts to the silver electrode through the patterned window. The viscous polyimide residue removal requires an additional boiling or rinsing process in DI water stream, which causes layer delamination. In this paper, we invent a special process for polyimide residue removal process, which is the time-controlled dipping process into DI water at room temperature after the conventional polyimide wet etching process. We experimentally find 21-minute dipping into DI water is proper to remove the residue of the 50 um-thick polyimide support membrane after the conventional wet etching.

**Figure 8:**
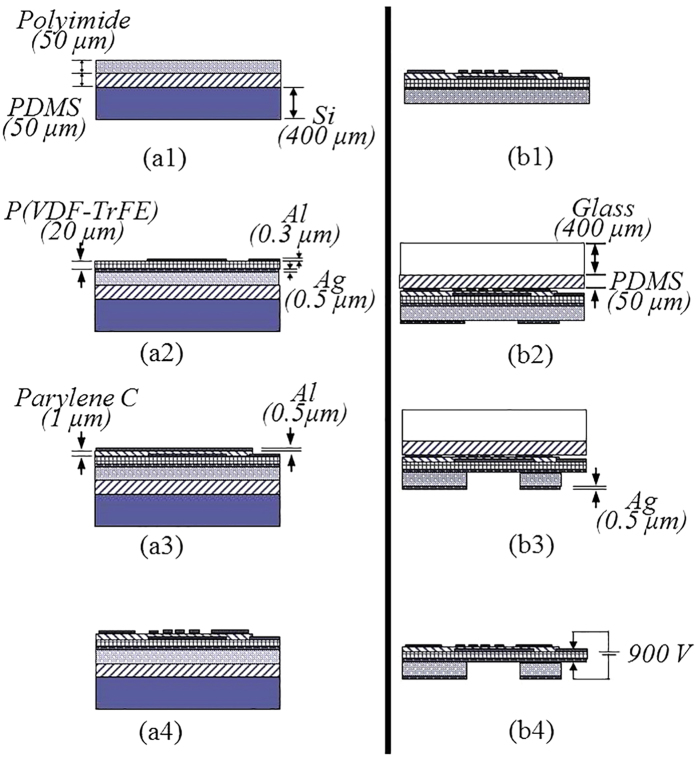
Fabrication process of the flexible human stress monitoring patch: (**a1**–**a4**) the multi-layer integration of skin temperature, skin conduce, and pulsewave sensors; (**b1**–**b4**) the windows fabrication process at the polyimide support membrane.

Figure 8 shows the fabrication process of the flexible human stress monitoring patch. The multi-layer integration of the three sensors is shown in Fig. 8a and the windows fabrication process using the polyimide residue removal process is introduced in Fig. 8b. The 50 μm-thick polyimide support membrane (Kapton® HN, Dupont™) is attached to a PDMS-coated silicon carrier wafer (see Fig. 8a1). 0.5 μm-thick silver electrode is thermally evaporated on the polyimide support membrane. The P(VDF-TrFE) solution is prepared to deposit the piezoelectric membrane on the silver electrode. The 75:25 P(VDF-TrFE) (Measurement Specialties, Inc.) pellets are dissolved in to Methyl Ethyl Ketone (MEK, Sigma Aldrich) solutions (35 wt%) at 60 °C using a sonicator. The dissolved solution is spin-coated on the silver electrode, followed by 2 hours curing at 130 °C to form a 20 μm-thick piezoelectric membrane. Then, a 0.3 μm-thick aluminum is thermally evaporated on the piezoelectric membrane and patterned to define piezoelectric poling and energy harvesting electrode using the AZ1512 etch mask (see Fig. 8a2). Instead of acetone, 50% diluted AZ400T (AZ electronic materials) aqueous solution^67^ is used to remove AZ1512 mask preventing damage on the P(VDF-TrFE) membrane. A 1 μm-thick parylene-C insulation layer is formed using chemical vapor deposition. The parylene-C insulation layer is patterned using reactive ion etching (RIE) followed by patterning a thermally evaporated 0.5 μm-thick aluminum shadow mask on the insulation layer. (see Fig. 8a3). The aluminum shadow mask is patterned again to define the skin temperature sensor and the skin conductance sensor (see Fig. 8a4).

For the windows fabrication on the other side of the patch, the PDMS-coated silicon carrier wafer is detached from the multi-layer structure of the polyimide support membrane, the piezoelectric membrane, the insulation layer, and the electrode layer (see Fig. 8b1). A transparent PDMS-coated glass carrier wafer is attached to the skin conductance and the skin temperature sensors side of the detached multi-layer structure. Then, a 0.5 μm-thick silver is thermally evaporated on the polyimide support membrane and patterned to define a sliver shadow mask for the windows formation on the polyimide support membrane (see Fig. 8b2). The windows are formed by 20 minutes chemical wet etching using the etchant of KOH-ethanol-DI water (1:8:2) mixture at 70 °C followed by the previously described polyimide residue removal process with 21 minutes of dipping condition, thereby defining the multi-purpose silver interlayer electrode without damage (see Fig. 8b3). The multi-layer structure having the windows on the polyimide support membrane is diced and released from the PDMS coated glass wafer. The P(VDF-TrFE) membrane is poled by supplying 900 V electric voltage across the silver and the aluminum electrodes at 90 °C for 30 minutes. The fabricated stress monitoring patch is as small as 24.7 mm × 15.0 mm × 70 μm with the skin contact area of 3.71 cm^2^ and the weight of 49.5 mg.

## Additional Information

**How to cite this article**: Yoon, S. *et al.* A Flexible and Wearable Human Stress Monitoring Patch. *Sci. Rep.* **6**, 23468; doi: 10.1038/srep23468 (2016).

## Supplementary Material

Supplementary Information

## Figures and Tables

**Figure 1:**
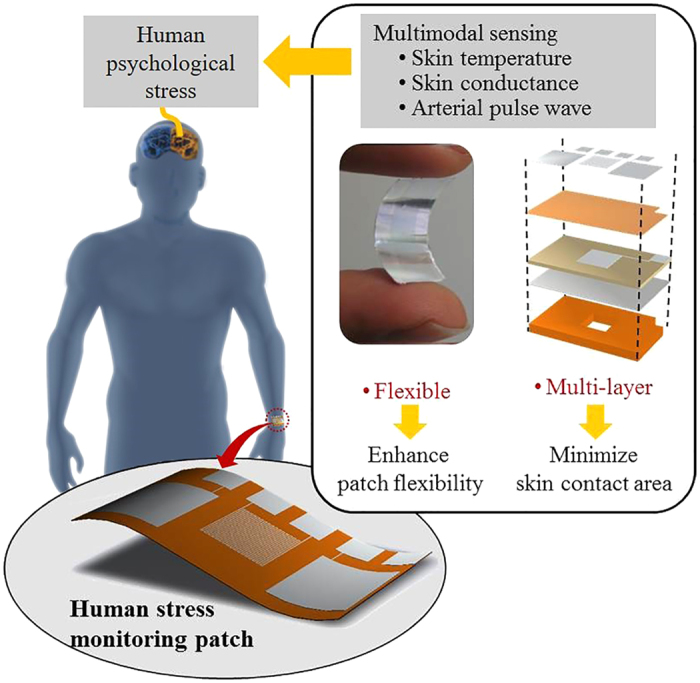
A flexible human stress monitoring patch, composed of multimodal sensors for daily use.

**Table 1:** Performance of the skin temperature sensor, the skin conductance sensor, and the pulsewave sensor in the fabricated flexible human stress monitoring patch.

Measurand	Skin temperature	Skin conductance	Pulsewave
Range	30–40 °C	2–20 μS	50–220 BPM, 40–120 mmHg
Nonlinearity	0.021%	0.447%	–
Resolution	–	≤0.02 μS	–
Sensitivity	0.31 Ω/°C	0.28 μV/0.02 μS	35 μV/mmHg
Time delay	–	–	70.00 ± 7.42 msec

## References

[b1] Mann J. & Currier D. “Stress, genetics and epigenetic effects on the neurobiology of suicidal behavior and depression”, Eur. Psychiat. 25(5), 268–271 (2010). 10.1016/j.eurpsy.2010.01.009 PMC2896004 20451357

[b2] Macan T., Shahani C., Dipboye R. & Philips A. “College students’ time management: correlations with academic performance and stress”, J. Educ. Psychol. 82(4), 760–768 (1990).

[b3] Pickering T. & Thomas G. “Mental stress as a causal factor in the development of hypertension and cardiovascular disease”, Curr. Hypertens. Rep. 3(3), 249–254 (2001). 11353576 10.1007/s11906-001-0047-1

[b4] Hans S. Stress in health and disease Ch. 4, 725–895 (Butterworth-Heinemann, 1976).

[b5] Adams P. *et al.* “Towards personal stress informatics: comparing minimally invasive techniques for measuring daily stress in the wild”, *Proceedings of the 8*^*th*^ *International Conference on Pervasive Computing Technologies for Healthcare*, Oldengurg. Brussels: ICST (doi: 10.4108/icst.pervasivehealth.2014.254959) (2014, March 20–23).

[b6] Horowitz M., Wilner N. & Alvarez W. “Impact of event scale: a measure of subjective stress”, Psychosom. Med. 41(3), 209–218 (1979). 472086 10.1097/00006842-197905000-00004

[b7] Lee J., Hwang Y., Cheon K. & Jung H. “Emotion-on-a-chip (EOC): evolution of bio chip technology to measure human emotion using body fluids”, Med. Hypotheses 79, 827–832 (2012). 23036904 10.1016/j.mehy.2012.09.002

[b8] Healey J. A. & Picard R. W. “Detecting stress during real-world driving tasks using physiological sensors”, IEEE Trans. Intell. Transp. Syst. 6(2), 156–166 (2005).

[b9] Cacioppo J. Handbook of psychophysiology 3rd edn (eds Tassinary L. *et al.*) Ch. 7, 159–181 (Cambridge, New York, 2007).

[b10] Carlson N. Foundation of physiological psychology 3rd edn Trans. Kim H. *et al.* Ch. 11, 459–466 (Sigmapress, 1998).

[b11] Kreibig S., Wilhelm F., Roth W. & Gross J. “Cardiovascular, electrodermal, and respiratory response patterns to fear- and sadness-inducing films”, Psychophysiology 44, 787–806 (2007). 17598878 10.1111/j.1469-8986.2007.00550.x

[b12] Porges S. “Vagal tone: a physiologic marker of stress vulnerability”, Pediatrics 90(3), 498–504 (1992). 1513615

[b13] Karthikeyan P., Murugappan M. & Yaacob S. “A review on stress inducement stimuli for assessing human stress using physiological signals”, *IEEE 7*^*th*^ *International Colloquium on Signal Processing and its Applications (CSPA)*, Penang. IEEE. (doi: 10.1109/CAPA.2011.5759914)(2011, March 4–6).

[b14] Dooren M., Vries J. & Janssen J. “Emotional sweating across the body: comparing 16 different skin conductance measurement locations”, Physiiol. Behav. 106, 298–304 (2012). 10.1016/j.physbeh.2012.01.020 22330325

[b15] Kushki A., Fairley J., Merja S., King G. & Chau T. “Comparison of blood volume pulse and skin conductance responses to mental and affective stimuli at different anatomical sites”, Physiol. Meas. 32, 1529–1539 (2011). 21849720 10.1088/0967-3334/32/10/002 PMC5028198

[b16] Guo R. *et al.* “Pervasive and unobtrusive emotion sensing for human mental health”, *7*^*th*^ *International Conference on Pervasive Computing Technologies for Healthcare*, Venice. Brussels: ICST. (doi: 10.4108/icst.pervasivehealth.2013.252133)(2013, May 5–8).

[b17] Kim K., Bang S. & Kim S. “Emotion recognition system using short-term monitoring of physiological signals”, Med. Biol. Comput. 42, 419–427 (2004). 10.1007/BF02344719 15191089

[b18] Sun F. Mobile computing, applications, and services (eds Kuo C. *et al.*) 211–230 (Springer Berlin Heidelberg, 2012).

[b19] Chiang H. “Ecg-based mental stress assessment using fuzzy computing and associative petri net”, J. Med. Biol. Eng. 35, 833–844 (2015), doi: 10.1007/s40846-015-0095-7.

[b20] Kumar M. *et al.* “Stress monitoring based on stochastic fuzzy analysis of heartbeat intervals”, IEEE Trans. Fuzzy Syst. 20(4), 746–759 (2012).

[b21] Frese M. Psychological issues of human-computer interaction in the work place (Eds Ulich E. *et al.*) 163–181 (North-Holland Publishing Co., 1987).

[b22] Vrijkotte T., Doornen L. & Geus E. “Effects of work stress on abulatory blood pressure, heart rate, and heart rate variability”, Hypertension 35(4), 880–886 (2000). 10775555 10.1161/01.hyp.35.4.880

[b23] Lee Y., Lee B. & Lee M. “Wearable sensor glove based on conducting fabric using electrodermal activity and pulse-wave sensors for e-health application”, Telemed. E-Health 16(2), 209–217 (2010). 10.1089/tmj.2009.0039 20070159

[b24] Fletcher R. *et al.* “iCalm: wearable sensor and network architecture for wirelessly communicating and logging autonomic activity”, IEEE T. Inf. Technol. Biomed. 14(2), 215–223 (2010). 10.1109/TITB.2009.2038692 20064760

[b25] Knight J. *et al.* “The design of the sensvest”, Pers. Ubiquitous Comput. 9(1), 2005, pp. 6–19.

[b26] Quazi M., Mukhopadhyay S., Suryadevara N. & Huang Y. “Towards the smart sensors based human emotion recognition”, *Instrumentation and Measurement Technology Conference (I2MTC)*, Graz. IEEE. (doi: 1109/12MTC.2012.6229646)(2012, May 13–16).

[b27] Ark W., Dryer D. & Lu D. “The emotion mouse”, The 8^th^ International Conference on Human-Computer Interaction Munich. Lawrence Erlbaum (1999, August 22–26).

[b28] Choi J., Ahmed B. & Gutierrez-Osuna R. “Development and evaluation of an ambulatory stress monitor based on wearable sensors”, IEEE T. Inf. Technol. Biomed. 16(2), 279–286 (2012). 10.1109/TITB.2011.2169804 21965215

[b29] Brown L., Grundlehner B., Molengraft J., Penders J. & Gyselinckx B. “Body area network for monitoring autonomic nervous system responses”, Pervasive Computing Technologies for Healthcare 1–3 (2009).

[b30] Massot B., Baltenneck N., Gehin C., Dittmar A. & McAdams E. “EmoSense: an ambulatory device for the assessment of ANS activity-application in the objective evaluation of stress with the blind”, IEEE Sens. J. 12(3), 543–551 (2012).

[b31] Poh M., Swenson N. & Picard R. “A wearable sensor for unobtrusive, long-term assessment of electrodermal activity”, IEEE Trans. Biomed. Eng. 57(5), 1243–1252 (2010). 20172811 10.1109/TBME.2009.2038487

[b32] Tanev G., Saadi D., Hoppe K. & Sorensen H. “Classification of acute stress using linear and non-linear heart rate variability ananlysis derived from sternal ecg”, *Conf. Proc. IEEE Eng. Med. Biol. Soc.* Chicago. IEEE. (doi: 10.1109/EMBC.2014.6944349)(2014, August, 26–30). 10.1109/EMBC.2014.6944349 25570717

[b33] Fernandez C. *et al.* “Physiological responses induced by emotion-eliciting films”, Appl. Psychophysiol. Biofeedback 37, 73–79 (2012), doi: 10.1007/s10484-012-90180-7. 22311202

[b34] Atterhog J., Eliasson K. & Hjemdahl P. “Sympathoadrenal andcardiovascular responses to mental stress, isometric handgrip, and cold pressor test in asymptomatic young men with primary t wave abnormalities in the electrocardiogram”, Br. Heart J. 46, 311–319 (1981). 7295424 10.1136/hrt.46.3.311 PMC482650

[b35] Parsons K. Human thermal environments: the effects oh hot, moderate, and cold environments on human health, comfort, and performance 3rd edn, Ch. 3 (CRC press, 2014).

[b36] Hjortskov N. *et al.* “The effect of mental stress on heart rate variability and blood pressure during computer work”, Eur. J. Appl. Physiol. 92, 84–89 (2004). 14991326 10.1007/s00421-004-1055-z

[b37] Samuel S., Manuel G., Manuel M., Javier V. & Edir D. “Heart rate variability during high-intensity exercise”, J Syst Sci Complex 26, 104–116 (2013).

[b38] Taelman J. & Vandeput S. “Instantaneous changes in heart rate regulation due to mental load in simulated office work”, Eur J Appl Physiol 111, 1497–1505 (2011). 21188414 10.1007/s00421-010-1776-0

[b39] Gil E. *et al.* “Photoplethysmography pulse rate variability as a surrogate measurement of heart rate variability during non-stationary conditions”, Physiol. Meas. 31, 1271–1290 (2010). 20702919 10.1088/0967-3334/31/9/015

[b40] Msc M., Yamasaki M., Sasaki T. & Nakayama H. “Fall in skin temperature of exercising man”, Br. J. Sports Med. 26(1), 29–32 (1992). 1600450 10.1136/bjsm.26.1.29 PMC1478977

[b41] Kim D., Kim J., Lee E., Whang M. & Cho Y. “Interactive emotional content communications system using portable wireless bifeedback device”, IEEE Trans. Consum. Electron. 57(4), 1929–1935 (2011).

[b42] Tseng H., Tian W. & Wu W. “Flexible PZT thin film tactile sensor for biomedical monitoring”, Sensors 13(5), 5478–5492, doi: 10.3390/s130505478 (2013). 23698262 PMC3690010

[b43] Li C. *et al.* “Flexible dome and bump shape piezoelectric tactile sensors using PVDF-TrFE copolymer”, J. Microelectromech. Syst. 17(2), 334–341 (2008).

[b44] Kim D., Roh H., Kim Y., Kim Y. & No K. “Selective current collecting design for spring-type energy harvester”, RCS Adv. 5, 10662–10666, doi: 10.1039/C4RA16443A (2015).

[b45] Ren G., Cai F., Li B., Zheng J. & Xu C. “Flexible pressure sensor based on a P(VDF-TrFE) nanofiber web”, Macromol. Mater. Eng. 298, 541–546 (2013).

[b46] Sharma T., Je S., Gill B. & Zhang J. “Patterning piezoelectric thin film PVDF-TrFE based pressure sensor for catheter application”, Sens. Actuator A-Phys. 177, 87–92 (2012).

[b47] Ohigashi H. *et al.* “Piezoelectric and ferroelectric properties of P(VDF-TrFE) copolymers and their application to ultrasonic transducers”, Ferroelectrics 60, 263–276 (1984).

[b48] Xiao S., Che L., Li X. & Wang Y. “A novel fabrication process of MEMS devices on polyimide substrate”, Microelectron. Eng. 85, 452–457 (2008).

[b49] Dobrzynska J. & Gijs M. “Flexible polyimide-based force sensor”, Sens. Actuator A-Phys. 173, 127–135 (2012).

[b50] Vaillancourt J. *et al.* “All Ink-jet-printed carbon nanotube thin-film transistor on a polyimide substrate with an ultrahigh operating freaquency over 5 GHz”, Appl. Phys. Lett. 93, 243301, doi: 10.1063/1.3043682 (2008).

[b51] Kwon H., Kim J. & Choi W. “Development of a flexible three-axial tactile sensor aray for a roboric finger”, Microsyst. Technol. 17, 1721–1726 (2011).

[b52] Choi W. “Polymer micromachined flexible tactile sensor for three-axial loads detection”, Trans. Electr. Electron. Mater. 11(3), 130–133 (2010).

[b53] Sim J., Youn S. & Cho Y. “A thermal peripheral blood flowmeter with contact force compensation”, J. Micromech. Microeng. 22, 125014, doi: 10.1088/0960-1317/22/12/125014 (2012).

[b54] Platini P. “Blood pressure behaviour during physical activity”, Sports Med. 5(6), 353–374 (1988). 3041529 10.2165/00007256-198805060-00002

[b55] Yoo B. *et al.* “Anatomical consideration of the radial artery for transradial coronary procedures: arterial diameter, branching anomaly and vessel tortuosity”, Int. J. Cardiol. 101, 421–427 (2005). 15907410 10.1016/j.ijcard.2004.03.061

[b56] Hibbleler R. Mechanics of Materials 8th edn Ch. 12, 569–579 (Prentice Hall, 2011).

[b57] Knight J. F. & Baber C. “A tool to assess the comfort of wearable computers”, Hum. Factors 47(1), 77–91 (2005). 15960088 10.1518/0018720053653875

[b58] Cho G., Lee S. & Cho J. “Review and reappraisal of smart clothing”, Int. H. Hum.-Comput. Interact. 25(6), 582–617 (2009).

[b59] Chuo Y. *et al.* B. “Mechanically flexible wireless multisensor platform for human physical activity and vitals monitoring”, IEEE Trans. Biomed. Circuits Syst. 4(5), 281–294 (2010). 23853374 10.1109/TBCAS.2010.2052616

[b60] Akhmedov G., Shakov V., Trifel M. & Khanlarova A. “Corrosion resistance of aluminum alloys used in hydraulic construction”, Hydrotechnical Construction 2(3), 215–218 (1968).

[b61] Choi H., Hong S., Sung T. & No K. “Effects of surface morphology on retention loss of ferroelectric domains in poly(vinylidenefluoride-co-trifluoroethylene) thin films”, Appl. Phys. Lett., 99, 092905, doi: 10.1063/1.3632042 (2011).

[b62] Lee C. & Zhang Y. “Reduction of motion artifacts from photoplethysmographic recordings using a wavelet demoising approach”, *Biomedical Engineering, IEEE EMBS Asian-Pacific Conference on*, Kyoto. IEEE. (doi: 10.1109/APBME.2003.1302650)(2003, October, 20–22).

[b63] Kim B. & Yoo S. “Motion artifact reduction in photoplethysmography using independent component analysis”, IEEE Trans. Biomed. Eng. 53(3), 566–568 (2006). 16532785 10.1109/TBME.2005.869784

[b64] Reddy K., George B. & Kumar J. “Motion artifacet reduction and data compression of photoplethysmo-graphic signals utilizing cycle by cycle fourier series analysis”, *IEEE International Instrumentation and Measurement Techonology Conferences*, Victoria. IEEE. (doi: 10.1109/IMTC.2008.4547026) (2008, May 12–15).

[b65] Reddy K. & Kumar J. “Motion artifact reduction in photoplethysmographic signals using singular value decomposition”, *Instrumentation and Measurement Technology Conference*, Warsaw, IEEE. (doi: 10.1109/IMTC.2007.379467)(2007, May 1–3).

[b66] Engel J., Chen J., Fan Z. & Liu C. “Polymer micromachined multimodal tactile sensors”, Sens. Actuator A-Phys. 117(1), 50–61 (2004).

[b67] Kim W. *et al.* “Patterning of ferroelectric poly(vinylidene fluoride-trifluoroethylene) film for nonvolatile memory devices”, Curr. Appl. Phys. 11, 341–344 (2011).

