# Supplementary material for: A Flexible and Wearable Human Stress Monitoring Patch
Source: Sci Rep. 2016 Mar 23;6:23468. doi: 10.1038/srep23468 (PMC4804278; doi:10.1038/srep23468)
Supplement: Supplementary Information [file srep23468-s1.pdf]

A Flexible and Wearable Human Stress Monitoring Patch

Sunghyun Yoon, Jai Kyoung Sim, and Young-Ho Cho*

NanoSentuating Systems Laboratory, Cell Bench Research Center
Korea Advanced Institute of Science and Technology (KAIST)
271 Daehak-ro, Yuseong-gu, Daejeon 305-701, Republic of Korea

E-mail: nanosys@kaist.ac.kr

Table S1 Time to reach the saturation temperature of the electrode by the skin temperature and external temperature.

External temperature / Skin temperature	0 ℃	10 ℃	20 ℃	30 ℃
30.0 ℃	22.0 msec	21.5 msec	21.0 msec	20.0 msec
32.5 ℃	33.0 msec	31.0 msec	29.5 msec	28.5 msec
35.0 ℃	41.0 msec	39.5 msec	38.0 msec	36.0 msec
37.5 ℃	47.5 msec	45.5 msec	43.5 msec	41.5 msec
40.0 ℃	52.5 msec	50.5 msec	48.5 msec	46.5 msec

Table S2 Electrode's saturation temperature by the skin temperature and the external temperature.

Skin temperature \ External temperature	0 ℃	10 ℃	20 ℃	30 ℃
30.0 ℃	30.0 ℃	30.0 ℃	30.0 ℃	30.0 ℃
32.5 ℃	32.5 ℃	32.5 ℃	32.5 ℃	32.5 ℃
35.0 ℃	35.0 ℃	35.0 ℃	35.0 ℃	35.0 ℃
37.5 ℃	37.5 ℃	37.5 ℃	37.5 ℃	37.5 ℃
40.0 ℃	40.0 ℃	40.0 ℃	40.0 ℃	40.0 ℃

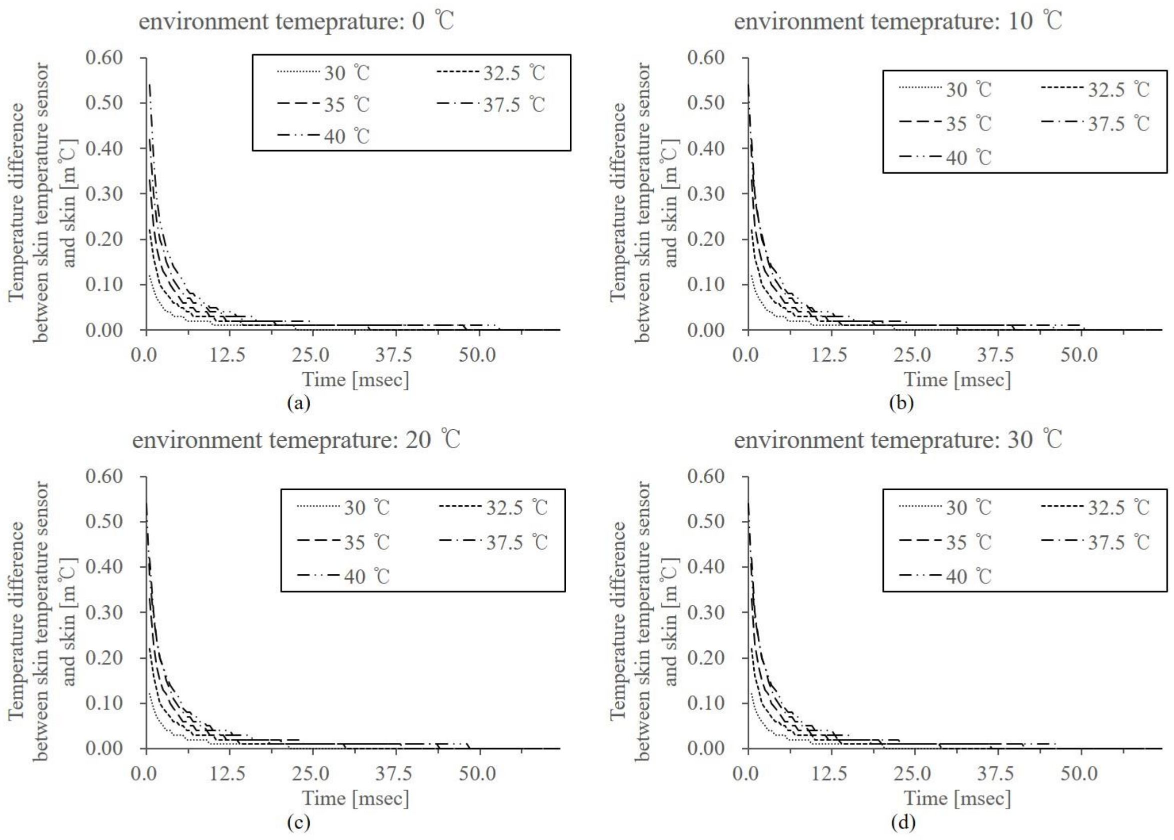

Figure S1 Time response of the temperature difference between the skin and the electrode at external temperature of (a) 0 ℃; (b) 10 ℃; (c) 20 ℃; (d) 30 ℃.
